# Eosinopenia as a diagnostic marker of bloodstream infection in a general internal medicine setting: a cohort study

**DOI:** 10.1186/s12879-020-4814-5

**Published:** 2020-01-30

**Authors:** Takanobu Hirosawa, Yukinori Harada, Kohei Morinaga, Hiroshi Takase, Michihiro Nin, Taro Shimizu

**Affiliations:** Department of Diagnostic and Generalist Medicine, Dokkyo Medical University Hospital, Clinical Education Building, Kitakobayashi 880, Mibu, Shimotsuga, Mibu, 321-0293 Japan

**Keywords:** Eosinopenia, Bloodstream infection, Blood culture

## Abstract

**Background:**

Little is known about the potential use of the eosinophil count as a predictive marker of bloodstream infection. In this study, we aimed to assess the reliability of eosinopenia as a predictive marker of bloodstream infection.

**Methods:**

This retrospective cohort study was performed in the outpatient department and general internal medicine department of a tertiary university hospital in Japan. A total of 189 adult patients with at least 2 sets of blood cultures obtained during the period January 1–December 31, 2018, were included; those with the use of antibiotic therapy within 2 weeks prior to blood culture, steroid therapy, or a history of haematological cancer were excluded. The diagnostic accuracies of each univariate variable and the multivariable logistic regression models were assessed by calculating the areas under the receiver operating characteristic curves (AUROCs). The primary outcome was a positive blood culture indicating bloodstream infection.

**Results:**

Severe eosinopenia (< 24.4 cells/mm^3^) alone yielded small but statistically significant overall predictive ability (AUROC: 0.648, 95% confidence interval (CI): 0.547–0.748, *P* < 0.05), and only moderate sensitivity (68, 95% CI: 46–85%) and specificity (62, 95% CI: 54–69%). The model comprising baseline variables (age, sex), the C-reactive protein level, and neutrophil count yielded an AUROC of 0.729, and further addition of eosinopenia yielded a slight improvement, with an AUROC of 0.758 (P < 0.05) and a statistically significant net reclassification improvement (NRI) (*P* = 0.003). However, the integrated discrimination index (IDI) (*P* = 0.284) remained non-significant.

**Conclusions:**

Severe eosinopenia can be considered an inexpensive marker of bloodstream infection, although of limited diagnostic accuracy, in a general internal medicine setting.

## Background

Blood cultures are necessary for the diagnosis and management of patients with bloodstream infection [1]. However, the usefulness of this test is limited except in special situations [e.g. inpatients with suspected infectious endocarditis [2] or meningitis [3]] because of its poor sensitivity in ambulatory outpatient [4], primary care, and hospital internal medicine department settings [5]. Additionally, many contaminants may lead to a false positive culture and, consequently, unnecessary therapy [6].

To date, no study has identified a highly sensitive and specific, easily measured, rapid, and inexpensive marker of bloodstream infection that correlates with infection severity and prognosis. Although the presence of chills [7], the C-reactive protein (CRP) level [8, 9], and the quick Sequential (Sepsis-Related) Organ Failure Assessment (qSOFA) score [10] have been identified as potential predictors of bloodstream infection, none has been determined to have adequate specificity and sensitivity.

Eosinopenia, defined as a reduced eosinophil count in peripheral blood, was previously identified as a good diagnostic marker of infection [11]. Although some studies reported that the absence of peripheral blood eosinophils could not be used as a clinically reliable marker of bacteraemia in a hospital inpatient setting [12, 13], those studies included limited numbers of patients and were not restricted to general internal medicine departments. Therefore, the potential usefulness of eosinopenia as a predictor of bloodstream infection in patients presenting or admitted to a general internal medicine department remains unclear. In this study we hypothesised that eosinopenia would be a reliable marker of bloodstream infection in adult patients treated in the general internal medicine department of a tertiary university hospital.

## Methods

### Study design and patient selection

This retrospective, single-centre cohort study included all consecutive in- and outpatients in the general internal medicine department, excluding intensive care unit and emergency department, of Dokkyo Medical University Hospital, Mibu, Tochigi, Japan, who underwent blood culture testing from 1 January to 31 December, 2018. Dokkyo Medical University Hospital is a tertiary teaching hospital. This study was conducted in accordance with the current version of the Declaration of Helsinki. The study protocol was approved by the institutional ethics committee of Dokkyo Medical University (No. R-20-18 J).

### Patient population

**Fig. 1:**
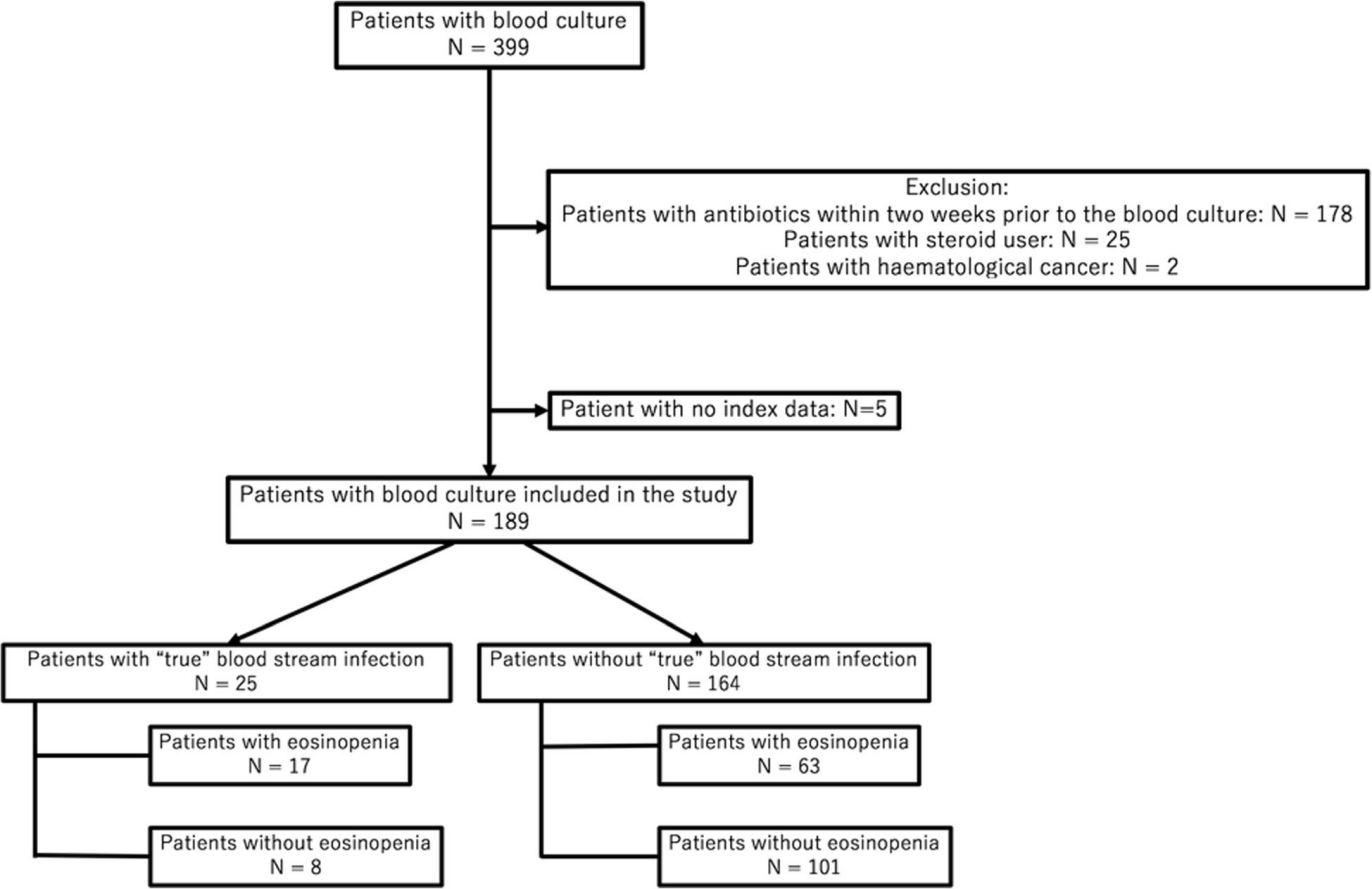
Flowchart of patient inclusion and exclusion in the study

From a total of 399 adult patients (age > 15 years) who underwent blood culture testing in the general internal medicine department during the study period, 205 were excluded because of antibiotic use within 2 weeks prior to the blood culture sampling (*n* = 178), steroid use (*n* = 25), or haematological cancer (n = 2). Five other patients were excluded because of a lack of data. The remaining 189 patients were enrolled in the study. A flow diagram of patient selection is shown in Fig. 1. All blood cultures were drawn at the discretion of the treating physician.

### Patient and public involvement

No patient involved.

### Outcome and definition

The primary study outcome was a positive blood culture indicative of bloodstream infection. We defined a bloodstream infection as the presence of a pathogenic microorganism in at least one blood culture bottle. Samples with bacterial contaminants were counted as negative cultures. The contamination criterion was the presence of multiplying coagulase-negative *Staphylococcus* species, *Bacillus* species, *Propionibacterium acnes* or *Corynebacterium* species in a single set of blood cultures. These bacteria were previously identified as frequent contaminants [14]. All such samples were excluded prior to the review of medical notes.

Absolute eosinopenia was differently defined in each research and does not have a universal definition [13, 15]. In this study, the optimal cut-off was defined as an eosinophil count of < 24.3 cells/mm^3^ from univariate analysis. The qSOFA, a recently developed measure for the rapid identification of infected patients at risk of mortality, was also applied [10, 16, 17]. This bedside clinical score identifies adult patients with suspected infection and a higher risk of poor outcomes typical of sepsis as those who meet with at least 2 of the following clinical criteria: respiratory rate of ≥22/min, altered mentation, or systolic blood pressure of ≤100 mmHg [10].

### Procedure

From each patient, the clinicians drew 10 mL of blood aseptically from a superficial vein and inoculated the sample into both aerobic and anaerobic cultures. They repeated the procedure using a different superficial vein to yield 2 sets of blood cultures for each patient [18]. The cultures were incubated in blood culture bottles containing BACTEC resin-beads (Bactec Plus Aerobic/23F and Anaerobic/22F bottles; Becton Dickinson Instrument Systems, Sparks, MD, USA). The bottles were incubated at 35 °C, sub-cultured daily, and inspected for bacterial growth for 6 days.

A fully automated BACTEC-FX blood culture incubation system (Becton Dickinson) was used to isolate bacteria from the blood cultures. Significant isolates were identified and tested for antimicrobial susceptibility according to the National Committee for Clinical Laboratory Standards guidelines [19]. All bacterial species isolated from blood culture bottles were confirmed using matrix-assisted laser desorption ionisation–time of flight mass spectrometry.

### Data collection

Patients’ medical records were reviewed to ensure that 2 attending clinicians considered the detected micro-organisms to be pathological, rather than contaminants. All data in this study were collected by the treating clinicians in the context of clinical management and included age, sex, presence of chills, and vital signs (mental status, respiratory rate, and systolic blood pressure) at the time of blood culture sampling. The potential markers of bloodstream infection assessed in this study included the serum CRP concentration and total white blood cell, neutrophil, and eosinophil count. All markers were measured within 1 day of blood culture sample collection. Eosinophil count was determined using an automated method.

### Analysis

Continuous variables are presented as medians and interquartile ranges [25th–75th percentiles] and were compared using the Mann–Whitney U test. Categorical or binary variables are presented as numbers (percentages) and were compared using the chi-squared test or Fisher’s exact test. The diagnostic accuracies of each univariate variable and the multivariable logistic regression models were assessed by calculating the corresponding area under each receiver operating characteristic curve (AUROC). A *P* value of < 0.05 was considered statistically significant. The 95% confidence intervals (CIs) were used to quantify uncertainty.

Previous studies identified the CRP level as a powerful predictive marker of bloodstream infection [11, 20]. In this study, we calculated the integrated discrimination index (IDI) and net reclassification improvement (NRI) [21] to assess whether the inclusion of eosinopenia into the model involving the baseline variables (age + sex) and CRP level would improve the predictive value. All statistical tests were performed using the R 3.6.0 and pROC package [22] for MacOS X (The R foundation for Statistical Computing, Vienna, Austria). Internal validation of the prediction models was conducted using ordinary nonparametric bootstrapping with 1000 bootstrap samples and bias-corrected, accelerated 95% CIs [23].

## Results

Of the 189 patients enrolled in the final analysis, 25 and 80 patients with a positive blood culture or eosinopenia, respectively, were identified during the study period. In 4 patients, multiple organisms were detected in the same blood culture specimen at the time of bloodstream infection diagnosis; 12 of the 25 identified bloodstream infections (48%) were due to Gram-positive organisms, while 9 (36%) were due to Gram-negative organisms. The baseline characteristics of infected and non-infected patients are shown in Table 1. Patients with a bloodstream infection had a significantly higher total white cell count and CRP concentration than those without a bloodstream infection. All other comparisons yielded insignificant results. Other patient characteristics that might have affected the eosinophil count [15] are presented in the Additional file 1: Table.S1.

**Table 1:** Comparison of characteristics between patients with and without bloodstream infection

Variable	Bloodstream infection (*n* = 25)	No bloodstream infection(*n* = 164)	*P* value*
Age, y (SD) [median]	71.8 (15.8) [75.0]	62.8 (20.0) [68.0]	0.246
Male, n (%)	12 (46)	93 (57)	0.582
CRP, mg/l [median, IQR]	120.5 [93.8, 50.4–160.7]	63.5 [40.4, 8.7–88.7]	0.017
Total white cell count, cells/mm^3^ [median, IQR]	11,360 [11,100, 8400-12,700]	9901 [8900, 6700-12,000]	0.009
Eosinophil count, cells/mm^3^ [median, IQR]	32.0 [11.0, 0.00–38.4]	115.1 [37.6, 0.00–100.3]	0.741
Neutrophil count, cells/mm^3^ [median, IQR]	10,141 [9601, 6969-11,842]	7971 [7250, 4754-9964]	0.075
qSOFA score 0–1	22	145	0.208
qSOFA score 2–3	3	19	
Chills, n (%)	8 (32)	34 (20)	0.891

*SD* standard deviation, *IQR* interquartile range, *CRP* C-reactive protein. *QSOFA* Quick Sequential (Sepsis-Related) Organ Failure Assessment

*P values by chi-squared, Mann-Whitney U test, or Fisher’s exact test

Table 2 presents the results of univariate analyses. Eosinopenia (AUROC: 0.648, 95% CI: 0.547–0.748, cut-off = 24.4 cells/mm^3^), neutrophil count (AUROC: 0.638, 95% CI: 0.519–0.758, cut-off = 9033 cells/mm^3^), and CRP concentration (AUROC: 0.699, 95% CI: 0.597–0.802, cut-off = 4.89 mg/dL) were all identified as significant predictive markers of bloodstream infection. Following bootstrapped multiple regression analysis (1000 bootstrap replicates), eosinopenia showed the same AUROC and 95% CI (AUROC: 0.648, 95% CI: 0.557–0.743). In contrast, white cell count (*P* = 0.185), qSOFA (*P* = 0.502), and presence of chills (*P* = 0.211) were not identified as statistically significant predictive markers. Further analysis revealed that eosinopenia could predict bloodstream infection with only moderate specificity (62, 95% CI: 54–69%) and sensitivity (68, 95% CI: 46–85%). The CRP concentration was more sensitive (80, 95% CI: 61–93%) but less specific (56, 95% CI: 48–64%). The neutrophil count was less sensitive (61, 95% CI: 41–80%) but more specific (69, 95% CI: 62–77%).

**Table 2:** Areas under the receiver operating characteristic curves of eosinophil, total white cell, neutrophil count, CRP, and qSOFA as potential markers of bloodstream infection identified through univariate analysis

Variable	Cut-off value	AUROC (95% CI)	P value*
Eosinophil count	< 24.4 cells/mm^3^	0.648 (0.547–0.748)0.648 (0.557–0.743)**	0.007
White cell count	> 10,950 cells/mm^3^	0.597 (0.472–0.723)	0.185
Neutrophil count	> 9033 cells/mm^3^	0.638 (0.519–0.758)	0.040
CRP, mg/l	> 4.89 mg/dl	0.699 (0.597–0.802)	0.001
qSOFA		0.502 (0.433–0.572)	0.952
Chills		0.556 (0.458–0.655)	0.211

*AUROC* Area under the receiver operating characteristic curve

*CI* confidence interval, *CRP* C-reactive protein

*QSOFA* Quick Sequential (Sepsis-Related) Organ Failure Assessment

*P values by chi-squared, Mann-Whitney U test, or Fisher’s exact test

**Bootstrapping method (1000 bootstrap replicates)

**Fig. 2:**
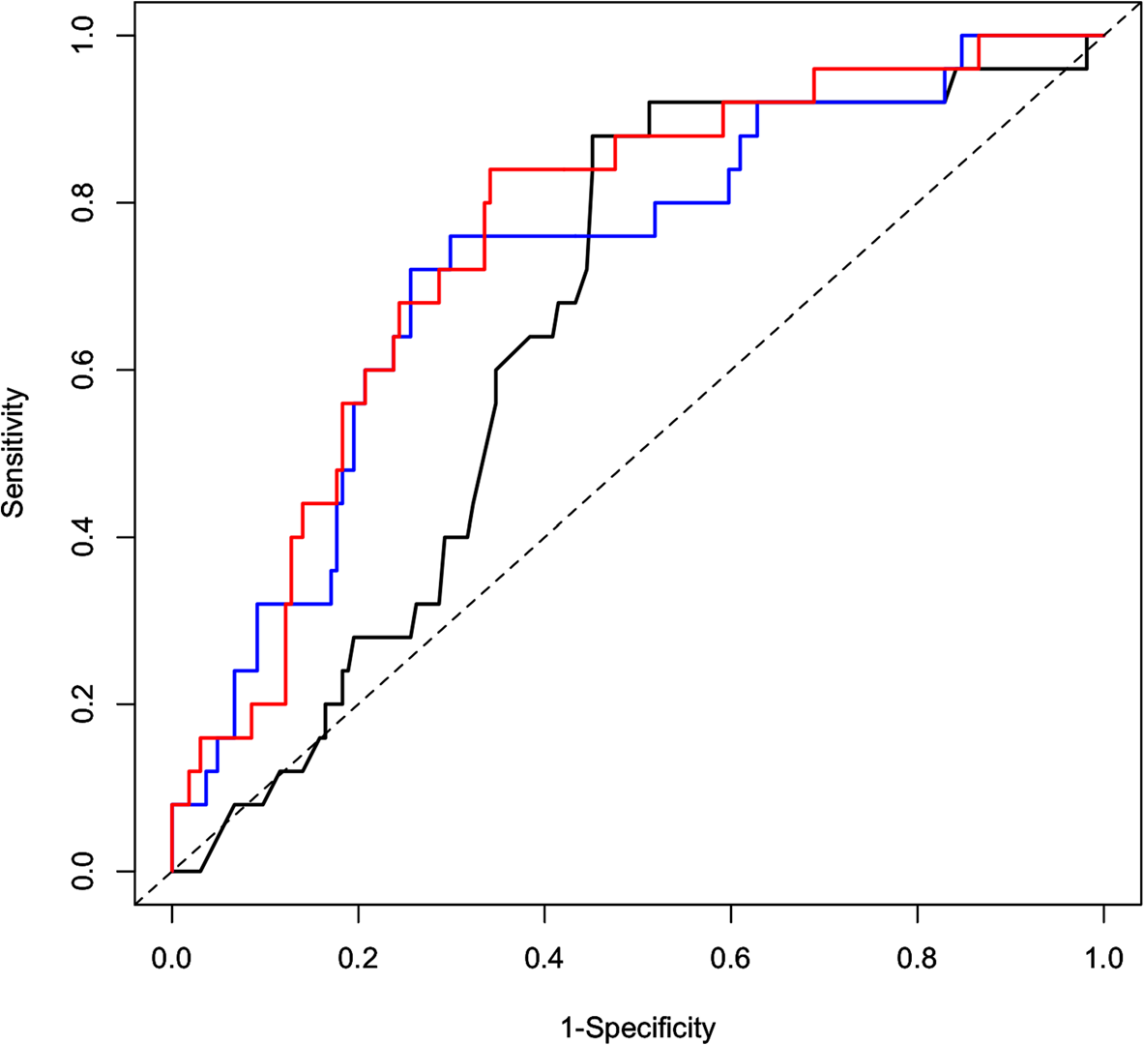
Areas under the receiver operating characteristic curves associated with bloodstream infection for the baseline variables alone (black line), baseline variables + C-reactive protein + neutrophil count (CRP, blue line), and baseline variables + CRP + neutrophil count + eosinopenia (red line)

Table 3 presents the AUROCs of the predictive models for bloodstream infection. The addition of CRP and neutrophil count to the baseline variables (age, sex) improved the AUROC (from 0.650 to 0.729; *P* = 0.002) and yielded a statistically significant IDI (*P* = 0.023) and NRI (*P* = 0.005). Further addition of eosinopenia to the model including the baseline variables, CRP, and neutrophil count led to a slight improvement, with an AUROC of 0.758 (*P* = 0.048) and a statistically significant NRI (*P* = 0.003). However, the IDI (*P* = 0.284) was not significant. Following bootstrapped multiple regression analysis, the model with eosinopenia showed the same AUROC. The corresponding ROC curves are shown in Fig. 2.

**Table 3:** Areas under the receiver operating characteristic curves of the predictive models for bloodstream infection

Model	AUROC (95% CI)	P value	IDI	P value	NRI	P value
Baseline variables*	0.650 (0.551–0.749)					
Baseline variables + CRP + neutrophil count	0.729 (0.622–0.835)	0.002**	0.069**	0.023**	0.583**	0.005**
Baseline variables + CRP + neutrophil count + eosinopenia	0.758 (0.664–0.853) 0.758 (0.667–0.845)****	0.048***	0.016***	0.284***	0.592***	0.003***

*AUROC* Area under the receiver operating characteristic curves

*CI* confidence interval, *CRP* C-reactive protein

*IDI* integrated discrimination index

*NRI* net reclassification improvement

* Including age, sex

** Compared with the model with baseline variables

***Compared with the model with baseline variables + CRP + neutrophil count

****Bootstrapping method (1000 bootstrap replicates)

## Discussion

According to our findings, eosinopenia alone yielded a reasonable overall predictive ability, but only moderate sensitivity and specificity for bloodstream infection in a cohort of patients who presented or were admitted to the department of general internal medicine at our university hospital. However, we found that eosinopenia was a more useful predictor of bloodstream infection than the qSOFA score and presence of chills in general internal medicine setting, excluding intensive care unit and emergency department. Moreover, the inclusion of eosinopenia in the prediction model comprising the baseline variables and CRP led to a slight improvement in the AUROC. These results suggest that eosinopenia may be useful as an inexpensive predictor of bloodstream infections. However, further investigations would be needed to exclude bloodstream infection.

Our study can be distinguished from previous work by a notable strength, namely the collection of data from a general internal medicine department. Although chills or qSOFA were previously identified as useful predictors of bloodstream infection in an intensive care unit or emergency department setting [11, 20, 24], our study showed that neither factor was a significant predictor of bloodstream infection in our general internal medicine department. This inconsistency suggests that chills and qSOFA may only be useful predictors in patients with a severe acute condition, who often present to an emergency department or are admitted to intensive care unit, but not in patients with milder conditions who would present to a general internal medicine department. Additionally, our finding that eosinopenia is a predictor of bloodstream infection suggests that this marker may be a useful tool for predicting such infections in patients with a mild general condition. We found that the addition of an elevated CRP level and elevated neutrophil count to the baseline variables yielded a stronger predictive measure. Further addition of severe eosinopenia to this model also led to a slight improvement in the predictive ability, even though the eosinophil count itself was not a sufficiently predictive marker.

This study had several limitations. First, it was conducted in a single department at a single centre, and therefore, our results cannot be easily generalised to the intensive care unit, surgery, and emergency department. Second, we excluded 178 patients (44.6%) who used antibiotics within 2 weeks prior to blood culture sampling, which may pose a risk of inducing a selection bias. This may be due to the tertiary nature of our institution, as patients with bloodstream infection may have initially visited a primary clinic and began to receive antibiotic treatment prior to referral to our department. Third, no clear criteria have been set to determine which patients would be subjected to blood culture. Rather, this decision was made by the treating physician on a case-by-case basis. Fourth, we excluded patients with haematological diseases, eosinophilia, and steroid users, as such cases are rarely seen in our department. Accordingly, our findings are not generalisable to these patient groups or areas with a high prevalence of these diseases. Fifth, not only severe and shaking but also mild to moderate chills were included in this study. According to the original article, the more severe degree of chills, especially shaking chills, suggests the high risk of bacteremia [7]. That could explain our insignificant results. Finally, procalcitonin was recently identified as a novel predictive marker of bloodstream infection, although one study identified it as a poor predictor of culture positivity [25]. In our study, procalcitonin was not evaluated in most cases. The results of our AUROC analysis suggest that when eosinopenia was modelled as a continuous variable, its diagnostic utility as a marker of bloodstream infection was limited.

## Conclusion

In summary, severe eosinopenia can be considered an inexpensive marker of bloodstream infection, although of limited diagnostic accuracy, in a general internal medicine setting.

## Supplementary information

**Additional file 1.** Additional characteristics of patients who underwent blood culture.

## Data Availability

The datasets generated and/or analysed in this study are not publicly available because it is possible that individual anonymity could be compromised.

## Abbreviations

AUROCs: Areas under the receiver operating characteristic curves
CRP: C-reactive protein
IDI: Integrated discrimination index
NRI: Net reclassification improvement
QSOFA: quick Sequential (Sepsis-Related) Organ Failure Assessment
