# Supplementary material for: Eosinopenia as a diagnostic marker of bloodstream infection in a general internal medicine setting: a cohort study
Source: BMC Infect Dis. 2020 Jan 30;20:85. doi: 10.1186/s12879-020-4814-5 (PMC6990586; doi:10.1186/s12879-020-4814-5)
Supplement: Supplementary file 1 — Additional file 1. Additional characteristics of patients who underwent blood culture. [file 12879_2020_4814_MOESM1_ESM.pdf]

Variable	Bloodstream infection (n = 25)	No bloodstream infection (n = 164)
Autoimmune	0	6
Asthma	2	4
Pancreatitis	0	2
Cytotoxic chemotherapy	0	2
Trauma	0	0
Recent surgery	0	0
Gastrointestinal bleeding	0	0
Rhinitis	0	0
Atopic dermatitis	0	0
